# The Clinical Diagnosis of Bright’s Disease

**Published:** 1883-01

**Authors:** C. W. Purdy

**Affiliations:** Chicago


CHICAGO MEDICAL

JOURNAL & EXAMINER.

Vol. XLVI.—JANUARY, 1883.—No. 1.

### Original Communications.

### Article I.

The Clinical Diagnosis of Bright's Disease. By C. W. Purdy, M.D., Chicago.

To the careful student of pathology, the name of Bright's Disease, as applied to all that group of renal diseases characterized by the presence of albumen and tube casts in the urine, appears unscientific and inappropriate. The numerous and distinct pathological conditions of the kidneys, all giving rise to, or accompanied by albuminuria, and the various etiological factors leading thereto, would seem to indicate a generalization of pathology and causes too sweeping, in our days of scientific exactness, to be confined within the narrow limits of such a single term. It has hence been the custom with more recent writers to base the nomenclature of renal diseases on the pathological changes found in each case; and so minutely has this been attempted, that some confusion has arisen in the opposite extreme.

It is my purpose, in the present paper, to present the most practical facts and deductions afforded by careful clinical research, as we meet them day by day, rather than enter into even a synopsis of the voluminous literature on the minute pathology, and many abstruse theories connected with these morbid processes and their consequences. For purposes of familiarity, I shall retain the name of Bright's Disease as applied to what we know of chronic disease of the minute tubules of the kidneys, the intertubular fibrous tissues, and the small renal blood-vessels.

It is now fifty-five years since Bright published the first description of the morbid changes in these structures; or rather what he considered to be the various changes of one disease affecting these structures. This half century has been very fruitful in scientific discovery in this important field. To the foundations laid by the illustrious Bright, others have added, with the aid of chemistry and the microscope, a greater exactness and accuracy of pathological knowledge, and, as a consequence, a therapeutic success in cases which heretofore were deemed beyond the reach of science.

Not only this, but a startling array of diseases, some of the most perilous and fatal with which humanity is afflicted, slumbered in obscurity till recent investigation has demonstrated that albuminuria was the first link in the chain of their pathology.

Let us remember that the vast list of mortality arising from hæmorrhagic apoplexy, has for its cause, in the majority of instances, one that is now clearly traceable to renal lesion. Let the obstetrician bear in mind that the most alarming complication which he is destined to encounter, the *Eclampsia Gravidarum et Parturientium*, which strikes terror to the hearts of the firmest, is now known to be due, with scarcely an exception, to renal lesion. Again, what a vast army sleep beneath the sod, with the only scientific satisfaction that, chronicled with their names, is the old, respectable, orthodox epitaph, "*Died of Dropsy*," duly recorded in the death certificate. It is not too much to state that the majority of these succumbed to the results of some renal lesion.

Consider the very large number of acute inflammations within the chest, cardial, pleural and bronchial, occurring without assignable cause, and so rapidly destroying life, as a result of unsuspected renal fibrosis. These are instances of facts which surround us every day. Too often these lesions slumber unsuspectedly behind some trifling ailment, till some of the terrible effects of uræmia suddenly break forth in a vital organ, revealing the diagnosis only when it becomes too late to rescue the patient from impending death.

We perceive, from facts like these, the immense importance of an early diagnosis of these insidious renal lesions. Some of them are quite amenable to early treatment. Most of them are capable of therapeutical modification to the prolongation of life, when early recognized. All of them are well-nigh hopeless when discovered in their late stages.

It has seemed to me an unfortunate fact, that the whole range of organic renal diseases whose chief characteristic is albuminuria, have been looked upon by a large share of the profession as hopeless ones from the start. A patient, after ailing for some time, calls the attention of his physician to some swelling of his feet. The urine is examined, and found to contain a large amount of albumen and epithelial casts. Although the patient is comparatively young, with great natural resources, and as yet not seriously reduced to very decided anæmia, he is informed that he has Bright's disease, and his case is necessarily hopeless. Such is evidently a case of tubular nephritis not very far advanced, and quite amenable, in the majority of cases, to treatment. Numbers of such cases, under appropriate treatment, we now know are relieved of their dropsy, the urine rendered free from albumen and casts, and if they are not cured in the strictest sense, they at least live the usual length of life, and die of some other disease. I believe that many of those cases classed as parenchymatous nephritis, if recognized reasonably early, may be readily cured. Even in cases of renal fibrosis and amyloid disease, if only the diagnosis can be early established, many lives may be vastly prolonged, and many of the fatal complications often avoided by intelligent medication.

Assuming the above to be true, what an incentive it is to study with the utmost care the early recognition of these diseases. Some of them are quite easily diagnosed; so much so that they should not escape the attention of the merest amateur. But, on the contrary, the class of fibroses begin with insidiousness, and continue to quite an advanced stage of development, giving but few signs of warning, and these are only recognized by the diagnostician who is ever on the alert for them. It has been my duty to attend two of my medical brethren, both ripe in years and medical experience, neither of whom suspected any kidney disease whatever in their cases, till the complications of advanced granular atrophy revealed the treacherous nature of their disease; and in both these cases, they had undoubtedly labored unconsciously under this lesion for years.

Brilliant indeed have been the revelations of chemistry and the microscope in renal diseases, nor would I in the least detract from the laurels so justly won in these fields. But it requires no expert in chemistry or microscopy to diagnose the form and stage of the most important renal lesions. Careful clinical observation, always the most reliable source of medical knowledge, finds no exception to the rule here.

While the above is true, I would point out the frequent and gross error of resting the prognosis on too superficial an examination of the case. On the discovery of albumen in the urine, the patient is often practically abandoned to his fate. Let us push our inquiries further than this. Ascertain by careful inquiry if the patient has become anæmic, emaciated, and if prostration is marked. In other words, how long has the drain of serum been undermining the vital fluid? Next, are there any evidences that urea, abnormally retained in the blood, is threatening to explode on the cerebral, pulmonary, or other vital organs? Ascertain as near as possible the quantity, gravity, reaction and appearance of the urine, and approximately the amount of albumen it contains. By means of the microscope, some knowledge will be obtained from the various tube casts; yet remember that almost any form of cast due to kidney lesion may accompany any one special form of these lesions. Next, the age, history, constitution, and all important physical characteristics of the individual *per se*, should engage the attention. When all these have been duly considered, together with other symptoms presently to be mentioned, an intelligent estimate may be made of the existing lesion, its stage, whether or not the patient is threatened with immediate danger, and above all, what can be accomplished in the way of cure.

Having pointed out the prime necessity of an early diagnosis of renal lesions, we shall now call attention to the more important symptoms whereby we may more readily recognize each special morbid change.

### CHRONIC TUBAL NEPHRITIS.

*Parenchymatous Nephritis*, *Diffuse Nephritis*, as it is severally called, is pathologically known as the large white kidney. In its early stages, at least, it is essentially a catarrhal inflammation of the minute tubules of the kidney. Like inflammation in other mucous structures, there is increased denudation of epithelium, at first of the mucous lining of these tubules. Deprived to a greater or less extent of this epithelium, serum with albumen readily transudes through their walls. Hence, the ratio of albumen is always high in this form of kidney lesion, reaching at times 80 to 90 per cent. by bulk. The epithelium, exfoliated in large quantities, mixes with fibrine and chokes up the tubes, dilating them, and from the hydrostatic pressure behind, become packed into casts or moulds, some of which escaping, we find them in the urine as the characteristic cast (epithelial) of the lesion. As the disease progresses toward the advanced stages, increased numbers of these tubules become blocked up, as an increased area of the organ becomes involved, the pressure from behind dilating these tubes, general swelling is the result. The whole organ becomes enlarged to two or three times its normal size. If we follow the changes on to the last stages, we find a secondary contraction in many cases. This latter has led to much confusion in pathology as to the true nature of the original lesion. It is probable that most cases of primary tubal nephritis, if they last long enough, finally have superadded to them interstitial disease, and, as a consequence, secondary atrophy. It would indeed be unexpected, as a result of so much obstruction and stasis of fluids, and actual inflammation in the network of tubes, if the inter-tubular fibrous tissues did not at length participate in the change.

Parenchymatous nephritis of the chronic form, which is that to which we shall limit our discussion at present, may take its origin from acute nephritis, but in many cases, it begins insidiously, and without any acute preceding symptoms.

As to its etiology, constant exposure to cold and damp atmosphere, in the temperate zone, will be found the most common cause. The poison of scarlatina, and the hyperæmia consequent on frequent pregnancy, undoubtedly lead to it. It has a quite marked hereditary proclivity, often occuring in several members of the same family. Of the use of alcoholic beverages, in relation to which so much has been said, I do not think the proofs are conclusive enough to establish them among the frequent causes. In a large proportion of the cases, the disease concludes its whole course without any satisfactory cause becoming apparent.

In searching for the early indications of chronic nephritis, there are some symptoms, which, although vague, become of the utmost value if we are on the alert for them ; for they lead us at once to examine the urine, perhaps before œdema of the feet, or some more advanced symptom, reveals the character of the disease. Perhaps the most constant of these is a feeling of unaccountable weakness and lassitude. The patient eats well and sleeps well, but discovers that he is weak, and goes to a watering-place for a few weeks, only to return home unimproved in strength. The cause is due to the drain of albumen from the blood being greater than the food can replace.

The condition of the skin is important; always dry, with a husky feeling. These patients seldom perspire. The hair partakes of this dryness, and becomes thinned. Observe, in many such cases, the early indications of anæmia; not constant, it is true, but when present, it is important. If symptoms as above present themselves, even though there be no œdema, examine the urine. We observe, first, that the urine is much diminished in quantity. This symptom is never absent, so long as the nephritis continues to advance, or remains in progress. It varies, however, and keeps pretty constant pace with the activity and extent of the disease. It may be reduced to two or three ounces in twenty-four hours, and if so, we know that the kidneys are extensively involved. In the latter stages, it should be noted that if, as often happens, fibrosis is superadded, the urine may be equal in quantity to the normal amount, or even exceed this in some instances.

Next, we note that the urine is of very high color, so much so as sometimes to suggest its admixture with blood, and this is often the case. The urine is not transparent, but smoky and cloudy, containing *debris* of broken down tissues, epithelium, etc. The urinometer will show the urine to be usually high in specific gravity, between 1020 and 1030, corresponding inversely with the quantity of urine secreted.

The urine in tubal nephritis will always be found to contain albumen; this condition is never absent. It will be present in larger relative quantities in this than in any other kidney lesion, and so constantly true is this, that it becomes almost diagnostic of this lesion.

It is a most important aid to the practitioner to be able readily ty estimate the presence and quantity of albumen in a specimen of urine, and without which cases do not receive the comparison day by day so essential to estimate the progress of the disease. The old method, with heat and nitric acid, takes some time to accomplish, and minor accidents are frequent, as breaking of tubes, or the boiling over of the urine and acid; and moreover, if the amount of albumen is small, and the acid be added in excess, the albumen will be re-dissolved.

I have been in the habit, for several years, of estimating both the presence and approximate quantity of albumen by the method of Bödeker, as the quickest and most satisfactory. The process depends on the fact that albumen, in an acetic acid solution, is completely precipitated by ferrocyanide of potassium. I use a glass tube graduated to one hundred tenths of a drachm, which I fill with the urine to be tested. I then render the urine acid by the addition of a little strong acetic acid, and upon adding a few drops of the ferrocyanide of potass. solution, any and all the albumen present will be almost instantly precipitated. On setting the tube aside for a few hours, all the albumen settles to the bottom, and the percentage can be read off from the scale on the side of the tube, and compared, day by day, with sufficient accuracy for all practical purposes. To simply determine the presence of albumen, a small bottle of acetic acid and one of ferrocyanide of potassium solution may be carried in the pocket case. At the bedside of the patient, when necessary to ascertain the presence of albumen in suspected urine, simply call for a wine-glass, fill it partly full of the urine, add the acid and solution, and the desired information is instantly obtained. This method will be found most delicate and reliable, as I have satisfied myself from repeated experiments, although the method of Méhu is claimed to be the most delicate by the very high authority of Newbaur and Vogel.

The microscope will reveal the presence of epithelial casts in large numbers, usually in the early stage of nephritis; granular casts later; and still later we may find fatty casts, or all of these may be present. From the indications enumerated, we are in a position to diagnose positively if we are dealing with a case of nephritis, and also to form a correct estimate of its extent, and amenability to treatment.

If we are only on the alert always for these symptoms, we shall sometimes be so fortunate as to be able to make a diagnosis of nephritis, even before œdema, or some other grave symptom, announces it unmistakably, and thus still more fortunate in being able to prolong indefinitely or save our patient's life. There are other and more prominent symptoms of chronic nephritis, among which may be mentioned dropsy; and it is probable that this symptom is never absent if the nephritis lasts any extended length of time. Beginning ordinarily in the feet, face or hands, it usually becomes general, involving especially the subcutaneous connective tissue. In some cases the swelling is enormous; so great, in fact, that rupture of the skin takes place, and the serum escapes in large quantities. The serous cavities frequently become the seat of effusion; in fact, almost constantly so in severe cases. Uræmia quite frequently occurs in this disease, but not so coustantly as in either acute nephritis or renal fibrosis. There are two reasons assigned for this less frequent appearance of uræmia in chronic tubular nephritis. One is that the appetite, for nitrogenous foods especially, is poor, and hence the source of production of urea is less operative. Perhaps the more probable reason is the storing up of large quantities of urea in the serum which occupies the large cavities and cellular tissues, and hence, not being present in the circulation in so large quantities at a time, is unable to reach the brain and other organs with damaging force. For the same reasons, probably, we do not encounter the acute chest affections, visual disturbances, etc., so frequently as in renal fibrosis. Hypertrophy of the left ventricle of the heart I believe to be rare in uncomplicated tubular nephritis. In the latter stages, however, when secondary contraction follows, it will be found quite common. Dyspepsia is frequent, due probably to serous infiltration into the gastric mucous tract.

### GRANULAR ATROPHY.

This brings us to the consideration of what is probably the most insidious and lurking form of disease to which the human frame is heir. Its appalling complications, when once they fall upon the patient, well-nigh banish the case from the domain of hope.

It is impossible to overestimate the stealthy manner in which this disease begins, and the silence with which it sometimes slumbers unsuspectedly for years. Nor is it possible to overestimate the terrible force and rapidity with which some of its complications destroy life, when once they explode with all their force upon some vital organ. A patient who has once fastened upon him renal fibrosis, from that minute literally hangs on the verge of a precipice, from which it will depend on the acutest powers of observation of his physician if he is rescued, or, indeed, even warned of his danger. Let us, then, study the landmarks and early signs whereby this terrible disease may be apprehended in its earliest manifestations.

*Granular atrophy*, *renal fibrosis*, or *interstitial nephritis*, as it is severally known, is essentially a chronic disease from its commencement. So far as the changes in the kidney are concerned, every step in the process is extremely gradual. Could we but limit the damage which this disease produces in the economy to the kidneys alone, we should have the satisfaction of prolonging immensely the life of the patient. This we cannot always do, but very much may be accomplished in warding off its threatening aspects, if only early discovered.

In searching for the earliest indications of renal fibrosis, a careful consideration of its etiological relations will frequently put us on our guard, even before there are any tangible symptoms developed. One of the most prominent and generally acknowledged causes of fibrosis is gout, first noted by Dr. Todd; and indeed, in England it is quite commonly called the gouty kidney. We find urate of soda deposited in the intertubular fibrous tissue in many cases of renal fibrosis, and previous history of gout is so common a precursor of the disease, as to leave no doubt of its prominence as a cause.

Of scarcely less prominence may be reckoned the absorption of lead, which Dickinson claims is responsible for more cases than any other known cause, ultimately destroying more than half of those whose lives are spent in paint and lead works.

Other less noted causes are valvular disease of the heart, gonorrhœa extending to the bladder, and perhaps malaria. Renal fibrosis has a strong hereditary proclivity. I believe that long-standing stricture of the urethra of small caliber is at least a predisposing cause.

Renal fibrosis is a disease mostly of middle or advancing age, unlike nephritis, which latter is most common in early life.

Among the first symptoms of interstitial disease will usually be found polyuria; the patient rises at night once, twice, or oftener, to pass his urine. This is due to the fact that more urine is secreted than normal, and the patient should be carefully interrogated on this point. It will be found, also, that the urine is of unusually light color, and free from deposits, as a rule.

Attacks of vertigo, without apparent cause, should arouse suspicion, and lead to inquiry into the state of the vascular system. Palpitation, and a feeling of want of breath, may further suggest examination of the heart, which is probably always hypertrophied in granular atrophy. The heart sounds are very loud, the impulse at the apex strong, and heaving, and there is a flat prolonged diastolic sound, heard loudest to the right of the sternum, between the insertions of the second and third right costal cartilages.

Hypertrophy of the left ventricle of the heart, which so constantly accompanies granular atrophy, has received much attention, owing to the important part it plays in the many grave complications of this disease. Many explanations of this interesting pathological change are on record, but none of them seem to me entirely satisfactory ; and it is probable that we do not yet know clearly the true causes of this change. Traube was the first to claim that it was due to increased blood pressure in the arterial circulation, consequent on the obliteration of the many arterioles in the kidney through advancing atrophy, and in this he is supported by Carl Bartels.

While I am satisfied that there is very decided increase of arterial blood pressure as a result of hypertrophy of the left ventricle, yet I cannot believe that the hypertrophy is due to any diminution of current in the renal vessels, for the following reasons: In chronic pulmonary tuberculosis, which has obliterated larger areas of vessels by far than are destroyed in atrophy of the kidneys, hypertrophy of the right heart does not follow; on the contrary, atrophy is frequently the attendant. Again, removal of the kidney surgically does not lead to hypertrophy of the left ventricle, nor does aneurism of the aorta; and lastly, hypertrophy of the left heart often begins early in fibrosis, before any considerable extent of the kidney becomes damaged.

Another explanation is, that owing to the accumulated waste products of tissues remaining in the circulation, due to defective action of the kidneys, the blood offers greater resistance to the arterioles and capillaries as it flows through them, and a more powerful ventricle is developed in order to overcome the increased resistance to the circulation. This would seem reasonable, were it not for the fact that both in parenchymatous disease and amyloid degeneration, there is the same retention of waste products in the circulation, and yet we seldom have hypertrophy in these lesions, unless granular atrophy becomes superadded to them. The explanation of Gull and Sutton is, that a general change in the whole vascular tubes is simultaneous with that in the kidneys, and part of the disease. As a consequence, the arteries partake of the fibroid degeneration, and lose their function of assisting the heart to a great extent in propelling the blood onward, and as a result, the heart compensates by developing a more powerful left ventricle. This would explain the early development of hypertrophy, and at the same time, the reason why it is confined to the lesion of granular atrophy.

Much information may usually be derived from the pulse in cases of renal fibrosis; in fact, it is frequently characteristic. It is described as yielding the impression of a "whip-cord," firm and rolling under the finger. It is very tense, quick in rhythm, and sharply defined. The carotids present much the same character, and may often be seen distinctly pulsating. In connection with symptoms referable to the vascular state, I have repeatedly noticed the occurrence of small aneurisms in superficial arteries, most frequently about the head, neck, and upper extremities.

It is not common, in the early stages of fibrosis, to find marked anæmia, and as a symptom it must be classed with those of the advanced periods of the disease. There is but little drain of serum albumen from the blood in these cases, and usually a good condition of digestion accompanies the early progress of the disease, thus easily compensating for what loss there is for some length of time.

Headache is a frequent and early accompaniment of fibrosis, sometimes extending over weeks, with short intermissions, and when no apparent cause exists for such constant headache, it should receive due consideration as a diagnostic.

A very annoying itching of the skin is often present in these cases, and sometimes becomes excessively tormenting to the patient. The skin is dry and rough, as in parenchymatous nephritis, and the patient perspires but seldom. Visual disorders occur very often, and are frequently the first apparent symptom the patient notices. These are of two kinds: one transient, and resulting from cerebral hyperæmia, owing to great fullness of the arterial circulation and powerful contraction of the left heart. The other often causes complete and permanent blindness, and is due to changes in the retina of the same character as we find in other tissues accompanying renal fibrosis.

Frequent and troublesome coryza is common as an early symptom, and it will be noticed that it is not much influenced by the usual remedies for that disease in its common form. Hæmorrhages from the mucous membranes, particularly that of the nose, are frequent. Uræmia of an acute form is often the first indication of the presence of fibrosis. It is frequently of an epileptic character, and it is probable that many cases of sudden death from supposed epilepsy in the first attack are due to granular atrophy.

In all such cases of first attack of supposed epilepsy, where there is no well-defined previous history of the disease, the symptoms should be most carefully scrutinized, and the urine examined. The convulsions, often extremely violent, are repeated at short intervals, which is not so frequently the case with true epilepsy. A decided urinous odor is frequently perceptible in the breath of such patients, and on inspection, crystals of urea may often be noticed upon the skin. Dickinson mentions, as a diagnostic of uræmia, that the pupils are dilated; while in convulsions from congestive causes they are contracted. I have satisfied myself, however, that this is not always to be relied on. There is another form of uræmia which is the natural termination of all cases of granular atrophy, if death does not overtake the patient through some other complication. This consists of a drowsiness, slowly merging into stupor and coma. If spasms are present, they are more clonic, and confined to certain sets of muscles.

There is a marked disturbance of the nervous system in early fibrosis, a feeling of uneasiness, referable to no particular cause, and frequently irascibility of temper. Dyspepsia is occasionally present in the early progress of fibrosis, accompanied by morning emesis and even vomiting, and especially as the disease advances, it is nearly always present to a greater or less extent.

As a rule, we have no warning dropsy, as in tubal nephritis, to put us on our guard. It is true, we may have some œdema of the feet or legs, but if so, it is usually in the advanced stages, and scarcely ever becomes prominent. A large proportion of the cases pass through this disease without any appearance of dropsy whatever.

If, in consequence of observing some of the above symptoms, it is determined to examine the urine, we first note that it is rather pale in color, and of light specific gravity, 1010 to 1015. This is the rule, but it sometimes varies. Crystals of uric acid, of large size, frequently become deposited, if the urine stands some time in a vessel or test tube. There is an increased quantity of the urine voided. In reference to albumen, this is usually present, though almost always in small quantities, averaging perhaps one-tenth that of nephritis. Albumen *may be* temporarily absent. If the patient is much reduced in muscular development, the appetite poor, and there be not much hypertrophy of the left ventricle, the amount of albumen will always be small. The character of the food, and the amount of exercise taken, also influence this symptom very much.

Tube casts of the pale hyaline kind are usually found to accompany early fibrosis, but, like albumen, may be temporarily absent. This absence is denied by some; but, making all due allowance for their scarcity and the difficulties in finding them, owing to their delicate outlines, and the properly adjusted illumination necessary to see them, still it is probable that they are sometimes absent in the early progress of fibrosis. In the latter stages, we may have present the dark granular casts.

Such, then, are the most reliable signs which accompany the early approach of granular atrophy. He who is the most minute observer, the most patient investigator; searching and probing every symptom to its source; going over his work repeatedly where the symptoms are obscure; comparing them from day to day, and from week to week if necessary; he alone will be rewarded by unveiling the secret haunts of this stealthy malady. Many of these early symptoms are readily referable by the patient and friends to other and minor causes, and it is their persistence, rather than threatening import, that leads him to consult his physician. Other and more grave symptoms occasionally accompany renal fibrosis quite early in the course of the disease. Hæmorrhagic apoplexy is a frequent and fatal one of these. "With blood-vessels weakened through altered nutrition, and often rendered brittle from atheroma; with redoubled power of the left ventricle, and with a blood much thinned from the renal drain on its fibrine;" it is no difficult matter to understand why this very serious complication is so frequent and so fatal. It unmasks at once the pathology and etiology lying behind the larger share of those cases of cerebral hæmorrhage with consequent paralysis or sudden death.

Pneumonia, pleurisy, pericarditis, and bronchitis of an acute and fatal character, frequently terminate the progress of renal fibrosis. The urine, being copious in quantity, is yet comparatively deficient in solid constituents, as indicated by its low gravity. The blood becomes in consequence surcharged with a large excess of nitrogenous excreta. A disposition to inflammatory attacks, especially in the above-named organs, is the result. When once inflammation sets in, it is fanned by increased arterial pressure, and a blood loaded with poisonous materials. Hènce the intractability and almost uniformly fatal result of these inflammations.

Emphysema, or œdema of the lungs, erysipelas, peritonitis, endocarditis, diarrhœa, vomiting of blood, hæmaturia, ascites, hydrothorax, and other symptoms, sometimes become a part of this far-reaching disease. They all deserve the most careful consideration in a comprehensive study of granular atrophy.

### AMYLOID DEGENERATION.

This name was first suggested by the apparent similarity of the characteristic resultant deposit to starch or cellulose. Lardaceous disease is also the name frequently applied to it, and this latter has received the sanction of the Pathological Society of London. We shall, however, retain the former name, as perhaps the most familiar, in our description of this diseased process.

Amyloid degeneration of the kidneys is in no sense a primary renal disease, but in all cases is a local manifestation of a general dyscrasia, expending its force principally on the smaller blood-vessels and abdominal lymphatics. The kidneys, while the most frequent, are but one of the favorite seats of this morbid process. The tendency is to excessive deposit of morbid matter, probably dealkalized fibrin, in the vessels of the kidneys, spleen, liver, bowels and other organs. Concerning a general cachexia, then, whose primary seat is probably in the blood itself, we may expect to find the most valuable diagnostic information by a careful study of its etiology. Among the causative factors of this disease, the most prominent is purulent suppuration. By far the majority of cases of amyloid disease of the kidneys will be found to follow chronic suppurative destruction of tissues elsewhere. Caries or necrosis of bone; phthisis with vomicæ; ulcerated cancer; abscesses of long standing; extensive bed-sores; in short, any suppurative disease, if exhaustive or of long standing, is exceedingly liable to lead to amyloid disease.

Bartels seems to think there is something in the nature of pus, which on absorption into the circulation, exerts a peculiarly damaging influence on the vessels, which leads to this degeneration. This may be so; yet it frequently happens that the evidences of amyloid disease do not become manifest till months or years have elapsed after the suppurative process has terminated.

I am inclined to believe that amyloid disease is rather a result of the general exhaustive effect of suppuration on the blood; as Dickinson expresses it, "an absence of the materials in the blood which go to make up pus," than the presence of the latter material in the circulation. This becomes more probable, when we consider that other chronic diseases whose tendency is to produce blood impoverishment, are also capable of giving rise to the amyloid degeneration. Syphilis, we find, is responsible for a large proportion of these cases. Its well-known tendency to produce anæmia, even in its early stages, indicates how profoundly it affects the vital fluid. Not only in its later complications of ulceration and necrosis, but before these lesions supervene, it is a quite common source of amyloid disease.

Scrofulous and tuberculous diathesis is considered to be a frequent cause of amyloid disease, resulting in a ratio of eight or ten per cent. of the cases. When we consider that these peculiar states of constitution have as their chief characteristic weak assimilation and diminished power of nutrition, and how nearly they are related to each other in many other respects; how often the tubercular disease springs from the strumous diathesis; how, as recent syphilographers tell us, tubercular disease is often the result of hereditary syphilis, we get a more comprehensive idea of the states of the system most prone to lead up to amyloid disease. It is probable that amyloid disease is due, not to any particular *materies morbus* in the blood exerting a poisonous influence on its vessels, but rather to a loss of balance between nutrition and waste, whereby the natural elements of the blood lose certain of their essential elements of nutrition, and a peculiar degeneration ensues, falling principally upon the vessels. It is immaterial whether this balance be destroyed through suppuration and excessive waste, or through defective nutrition or assimilation; they both conduce to the same result, for amyloid disease will be most often found to follow in the tracks of the one or the other.

The diagnosis of amyloid disease is not shrouded in as much obscurity as that of nephritis, nor by any means in as much as that of granular atrophy. For the most part clearly traceable to direct and appreciable causes to which it is secondary, it is also accompanied, and often preceded, by prominent symptoms in other organs which are not likely to escape attention.

From what has been said, it is clear that we would expect decided constitutional change, generally, before local symptoms point to amyloid kidneys. If tubal nephritis or granular atrophy sometimes attacks the kidneys of apparently healthy and even robust individuals, it is never so with amyloid disease. If there be no obvious chronic disease in progress, there is yet a marked cachectic appearance of the patient, pointing to an altered vital fluid from some underlying cause. The patient has a chronic anæmic appearance, a pale, yet muddy, hue of skin, and his movements indicate a sense of languor and weariness.

In reference to the indications which the urine itself presents; there is more or less frequency of micturition, and the quantity of urine voided is always large, especially in the early stages; usually more so than in fibrosis. Thirst is pretty constant, and œdema of the feet likewise, in the majority of cases. The latter may at first be transient, disappearing at night, to return during the day, especially if the patient be about on his feet. Diarrhœa is a most constant and annoying symptom, rarely, if ever, absent. It is usually accompanied by dyspepsia and often by vomiting. Diarrhœa returns again and again, and is one of the most frequent causes of death. These disturbances of the digestive tract are due, in the earlier stages, to excessive secretion of the mucous surfaces and a morbid deposit in the vessels, and in the worst form, to ulceration. General dropsy is the rule when the disease becomes developed. The thorax usually escapes, but ascites is pretty constant, and almost always general cellular œdema is present. The urine presents characters in some respects similar to those of fibrosis. As before remarked, it is pale, and of large quantity. Its specific gravity is even lower than in fibrosis. I have seen it reach 1004. Albumen is probably always present, and although it varies much as to quantity, as a rule, the latter is about midway between that of nephritis on the one hand, and fibrosis on the other. Unlike either interstitial or parenchymatous disease, in amyloid degeneration there is little diminution in the amount of urea excreted by the kidneys.

Casts are pretty uniformly present; mostly narrow fibrinous ones, but rarely are they numerous. The waxy and refracting casts are more common, especially in the later stages. Occasionally the broad casts found give a characteristic, dark-red reaction, when treated with iodine solution. Jürgens and Heschl, of Germany, and Cornil, of France, first called attention also to anilin-violet as a test for these casts. This strikes a red or pink color with amyloid material and some casts found in this disease. Some prefer this latter test to iodine, not only in detecting characteristic casts, but also pathologically, in testing amyloid deposits in the Malpighian tufts and renal vessels after death.

Enlargement of the spleen or liver or both very constantly accompany this disease. Precisely the same changes occur in the vessels here, as in the kidneys, and gives rise to the same pathological appearances and chemical reactions. It is said if tubercular disease of the lungs is in progress, when amyloid degeneration attacks the kidneys and liver, the former becomes arrested. I have verified this in one case.

I have observed two cases of diabetes mellitus, both of which were followed and accompanied by amyloid disease. Uremia occurs but rarely in the course of amyloid disease. It is probable that when it does so, that either nephritis or fibrosis has been superadded to the original malady. The same may be said of hypertrophy of the heart, rarely, if ever present, when it is so usually in the latter stages, and probably as a result of the addition of renal fibrosis.

As complications of amyloid disease; acute inflammations sometimes supervene, though with much less frequency than in any of the other forms of albuminuria. The serous inflammations are perhaps the most common, especially peritonitis. Pneumonia and pleurisy sometimes occur. Suppuration of the cellular tissue, and erysipelas sometimes result when the dropsy is extreme, and thrombosis of the femoral veins sometimes occurs from the same cause.

Such, then, are the more prominent clinical characteristics which accompany these lesions, grouped together under the term of Bright's disease. It must not be forgotten that any one form of lesion giving rise to its own particular sets of symptoms, may, and frequently does become complicated with one or the other, of those described. It is common, for instance, for a primary parenchymatous disease in its advanced stages to have engrafted upon it that of fibrosis, and perhaps equally common for the last named disease to follow that of amyloid disease. The inter-existence of these diseases has thrown much confusion into the diagnosis and clinical history of renal diseases, and calls for a careful differential study of the various symptoms.

First then, as to the history of the patient. If previously reduced by some chronic disease, as tuberculosis, scrofula, syphilis, or some lengthy suppurative process, it points strongly towards the amyloid form of disease. If the symptoms began in a vague protracted manner, extending over months, and the patient has a gouty history, or has pursued an occupation exposing him to influences of lead, it argues strongly for fibrosis. If again, the disease comes on after exposure to cold, or is not clearly traceable to other predisposing causes, it favors the presumption of nephritis. Age is an important consideration to note. Most cases of nephritis occur early in life; those of amyloid disease occur in early adult and middle life, while fibrosis for the most part is a disease of advancing age.

The urine, though subject to more or less variation in all cases, yet yields differential information the most valuable. Of albumen; if excessive in quantity, say 60 to 90 per cent by bulk, it indicates tubular disease; if smaller in quantity, say 20 to 50 per cent, it would be presumptive of amyloid degeneration; if extremely small in quantity, and temporarily absent, it is strongly indicative of fibrosis. The quantity of urine voided is much under the normal amount in nephritis; at least equal to, or somewhat more than normal in atrophy, and largely in excess in amyloid disease. The color is dark, and considerable sediment is present in nephritis; while in both amyloid disease and fibrosis, the color is light and very little deposits are present. The gravity of the urine in nephritis, is rather higher than normal; in atrophy, it is somewhat under the standard of health, and in amyloid lesions the specific gravity is decidedly below normal.

Of tube casts; the epithelial variety are said to be characteristic of nephritis, at least they are present in larger numbers than in any other lesion. Fibrous casts are most common in atrophy, and the waxy refracting casts are most prominent in amyloid disease. Casts giving a red reaction with iodine solution, are characteristic of the last named disease.

Dropsy is constant, and early in tubular disease, less so in the amyloid form, and exceptional in fibrosis.

Of the complications of these lesions, hypertrophy of the left ventricle of the heart is a marked presumptive of fibrosis, accompanying almost every case. It is less frequently present in either of the other forms, and when present in these it is mostly in their advanced stages.

Uræmia is very common in fibrosis, probably much less so in nephritis, and rare in amyloid disease. Much the same order obtains in relation to the various inflammatory complications, as well as the disorders of vision. They are all most common in fibrosis; quite frequent in nephritis, and rarely present in amyloid disease. Hæmorrhages of the various forms characterize atrophy. Enlarged liver and spleen, with obstinate diarrhœa are peculiar to amyloid degeneration. If there be any symptom predominently characteristic of nephritis, it must be accorded to that of dropsy.

Thus we find a very substantial ground-work for the basis of differential diagnosis of these lesions under most circumstances, when they comingle with each other in the advanced stages, and present to us aggregations of symptoms apparently anomalous and inseparable; if we but attentively study the early symptoms of each case with the necessary perseverence, we shall always find that in the inception of the malady its symptoms leaned unmistakably towards one or the other of these distinctive lesions. If, then, we trace the history onward, step by step, it will often become clear to us, the time, and form of a superadded lesion, till at length, what at first seemed a most perplexing conglomeration of symptoms, is in fact, but the expression of two independent pathological processes progressing side by side.

In cases of much obscurity in general symptomatology, if there is any rule more than another likely to elucidate the diagnosis of these lesions it is "the careful, repeated and systematic examination of the urine," so strongly insisted upon by Bartels. He who would know what is taking place in the kidneys, must thoroughly acquaint himself for weeks, and if necessary even months, with the character and contents of the urine. No single examination of this fluid so perpetually variable in health can possibly afford a comprehensive knowledge of its still more variable pathology, and he who studies these variations the most attentively through all the stages of these lesions is likely to become the most thorough master of the subject.

We urge a more thorough study of the earliest manifestations of these lesions, a research into their pre-clinical history, that they may more often become discoverable before they have advanced so far upon us as to largely compromise our therapeutical resources for their relief.

